# Linking Cellular Morphogenesis with Antifungal Treatment and Susceptibility in *Candida* Pathogens

**DOI:** 10.3390/jof5010017

**Published:** 2019-02-21

**Authors:** Jehoshua Sharma, Sierra Rosiana, Iqra Razzaq, Rebecca S. Shapiro

**Affiliations:** Department of Molecular and Cellular Biology, University of Guelph, Guelph, ON N1G 2W1, Canada; jehoshua@uoguelph.ca (J.S.); srosiana@uoguelph.ca (S.R.); irazzaq@uoguelph.ca (I.R.)

**Keywords:** *Candida*, Fungal morphogenesis, Antifungal drugs, Antifungal drug resistance, Fungal pathogens

## Abstract

Fungal infections are a growing public health concern, and an increasingly important cause of human mortality, with *Candida* species being amongst the most frequently encountered of these opportunistic fungal pathogens. Several *Candida* species are polymorphic, and able to transition between distinct morphological states, including yeast, hyphal, and pseudohyphal forms. While not all *Candida* pathogens are polymorphic, the ability to undergo morphogenesis is linked with the virulence of many of these pathogens. There are also many connections between *Candida* morphogenesis and antifungal drug treatment and susceptibility. Here, we review how *Candida* morphogenesis—a key virulence trait—is linked with antifungal drugs and antifungal drug resistance. We highlight how antifungal therapeutics are able to modulate morphogenesis in both sensitive and drug-resistant *Candida* strains, the shared signaling pathways that mediate both morphogenesis and the cellular response to antifungal drugs and drug resistance, and the connection between *Candida* morphology, drug resistance, and biofilm growth. We further review the development of anti-virulence drugs, and targeting *Candida* morphogenesis as a novel therapeutic strategy to target fungal pathogens. Together, this review highlights important connections between fungal morphogenesis, virulence, and susceptibility to antifungals.

## 1. Introduction to *Candida* and Candidiasis Infections

The fungal kingdom contains diverse organisms which make significant contributions to supporting life on our planet. Fungi are of vital importance to humanity as industrial manufacturers, play a key role in environmental nutrient recycling, and are model organisms for many eukaryotic processes. Our use of fungi throughout history has expanded in scope, from an edible agricultural resource, to the production of food, beverages and are a valuable source of antimicrobial compounds. However, there are also many fungal species that are serious pathogenic threats to plant, animal, and human health [1]. Currently, approximately 5 million fungal species have been classified, of which an estimated 300 are capable of establishing disease within a mammalian host [2,3]. As a human pathogen, it has recently been reported that, globally, fungal infections currently cause 1.5 million deaths per year [4], a statistic that rivals that of tuberculosis. Invasive fungal infections are a relatively modern problem, associated with newly-emerging populations of aging and immunocompromised individuals, such as HIV/AIDS patients, those undergoing cancer chemotherapy treatment, organ transplantation recipients, and others.

Amongst the most notable human-associated fungal pathogens are members from the *Candida* genus, including *Candida albicans, Candida tropicalis, Candida glabrata, Candida krusei, Candida parapsilosis*, and the emerging pathogens *Candida haemulonii* and *Candida auris* (Table 1); these fungi all cause a range of infections, which, particularly in immunocompromised patients, can lead to severe, invasive candidiasis including bloodstream infections [5,6,7]. Both acquired and inherited immunodeficiencies and other risk factors may predispose patients to *Candida* infections. The risk of candidiasis is increased in patients with underlying malignancies and those undergoing chemotherapy, patients undergoing hematopoietic stem cell or solid organ transplantation, those with immunosuppressive diseases (i.e., HIV/AIDS), patients using broad-spectrum antibiotics or corticosteroids and using invasive medical interventions (i.e., central vascular catheters), and those with certain genetic risk factors (i.e., impaired IL-17 immunity) [8,9,10,11]. In most cases, *Candida* spp. are opportunistic pathogens that reside as commensal organisms present in the body’s endogenous microbiome [12]. However, factors such as an impaired immune system, or implanted medical devices, can create an environment that enables invasive infections to arise [13]. Nosocomial infections of candidiasis are becoming more common, and account for more than 85% of all invasive fungal infections in both Europe and the United States [6]. Not only are these infections becoming more prevalent, they are accompanied by dangerously high mortality rates in both developing and developed countries alike. Globally, 30-day all-cause mortality rates range from 29 to 72% [14]. In 2016, Dio et al. [5] reported crude mortality rates of candidemia of 72.2% in Brazil, and Bongomin et al. [4] reported mortality rates in the UK in excess of 40%. 

There are currently almost 200 species of yeasts in the genus Candida [31], and the notable pathogens amongst them include *C. albicans*, *C. dubliniensis*, *C. parapsilosis*, *C. glabrata*, *C. tropicalis*, *C. lusitaniae*, and *C. krusei*. Additionally, *C. auris* has recently emerged as a highly multidrug-resistant pathogen, and a serious global health threat [32]. Amongst these *Candida* pathogens, *C. albicans* is the species mostly common associated with human infection [14]. Each of these *Candida* species possess critical factors that contribute to these fungi being well adapted as a pathogen to the human host. Processes such as cellular morphogenesis, cell-surface adhesion, phenotypic switching, biofilm formation, antifungal drug resistance, and secretion of hydrolytic enzymes are all well-established virulence mechanisms that aid in the success of these species as pathogens [33,34,35,36,37,38,39,40].

## 2. *Candida* Morphogenesis: Utility and Regulation

Certain *Candida* species possess the ability to undergo complex cellular morphological transitions; this trait is frequently linked to the virulence of these opportunistic pathogens [33,36,39,41]. There are three relevant morphotypes of *Candida* spp.: yeasts, hyphae and pseudohyphae, with hyphae and pseudohyphae sometimes collectively referred to as filamentous growth states [41,42]. Yeasts are ovoid, single cells that grow and divide via axial and bipolar budding, reminiscent of the the model organism *Saccharomyces cerevisiae* [43,44]. Hyphal states are indicative by their elongated state with a uniform width and parallel sides. They have pores in their septa that allow for cell to cell communication and tend to grow in a polarized manner [42,45]. Pseudohyphae share the traits of polarized growth and elongation with hyphae; however, they are ellipsoid and their septa are constricted. Pseudohyphae also tend to be phenotypically distinct from hyphae, via their highly branched nature, as their cells are smaller and take less time to divide [46]. While pseudohyphal growth is commonly observed amongst *Candida* species, the extent and frequency to which different species can undergo pseudohyphal growth, and the length and appearance of the pseudohyphae can vary significantly between species. Amongst *Candida* spp., only certain species possess the ability to undergo the morphological transition between yeast and filamentous growth states (Table 1). The best studied of these is *C. albicans*, which is readily able to transition between yeast, pseudohyphal, and hyphal growth states, under diverse environmental conditions [39]. Other *Candida* species, are similarly able to undergo a yeast to hyphal/pseudohyphal transition, including *C. tropicalis* [47] and *C. dubliniensis* [48]. The *Candida* species *C. parapsilosis*, *C. lusitaniae*, *C. haemulonii* and *C. krusei* are unable to form true hyphae, but can transition between yeast and pseudohyphal states [24,49,50,51]. Recently, a filamentous growth state was observed in the emerging *Candida* pathogen, *C. auris*, upon growth on NaCl-rich YPD, or upon deletion of the molecular chaperone *HSP90* [52,53]. Passage through a mammalian host also revealed a hyphal-like phenotype in *C. auris* [26]. This is an important finding, and one that may further help explain the success of *C. auris* as a highly virulent, emerging fungal pathogen. 

Cellular morphogenesis in *Candida* species between yeast and filamentous growth is often associated with virulence, with both yeast and filamentous forms contributing to pathogenesis in distinct capacities [54]. For *C. albicans*, it is suggested that the yeast morphology aids in fungal colonization, biofilm formation, and rapid dissemination into the host bloodstream [55]. On the other hand, the hyphal growth state facilitates deep tissue colonization, enhances biofilm formation, and enables macrophage evasion [45]. *C. albicans* cells in a hyphal morphology can invade epithelial cells by exerting a mechanical force to force growth between tissues, and similar mechanical stretching can enable hyphal escape from macrophages [56,57]. *C. albicans* hyphae also secrete candidalysin, a peptide toxin that is critical for virulence, via damage to host epithelial cell membranes, and stimulating host cell signaling pathways [58]. Candidalysin is itself derived from proteolytic cleavage of the Ece1 protein, the product of the hyphal-specific gene *ECE1* [58,59]. *C. albicans*’ thigmotropism also allows the pathogen to find weak points such as grooves in the host tissue to penetrate and cause further host damage [60]. 

Much experimental evidence has demonstrated that, when polymorphic *Candida* spp. are locked into one morphotype or the other, its virulence is lowered, or completely abolished [54,61,62,63]. This indicates that the specific functions demonstrated by each morphotype is directly responsible for the overall virulence of these polymorphic pathogens. Conversely, there are other species in the *Candida* genus that exist mainly in a yeast morphology, yet remain virulent. For example, *C. glabrata* is typically a non-dimorphic yeast that is capable of being highly virulent [17]. Additionally, while deleting genes involved in morphogenesis often results in loss of virulence, there are factors that are involved in morphogenesis and not virulence, and, conversely, virulence factors that play no apparent role in morphogenesis [54]. This suggests that, while filamentation and morphogenesis is an important virulence strategy for some *Candida* species, it is not universal, nor is it strictly required for virulence. There is a complex evolutionary relationship between fungal morphogenesis and virulence, which has been subject to in-depth exploration [63].

### 2.1. Key Signal Transduction Pathways That Govern Morphogenesis

The genes that promote *C. albicans* filamentation are tightly regulated by a network of key signaling pathways and transcription factors [64]. These signaling processes have been subject to significant research and review [39,64,65,66,67]. Briefly, these filamentous growth-promoting transcription factors are controlled by complex cellular signaling pathways that are ultimately triggered by a wide range of environmental signals, such as serum, nutrient limitation, temperature, pH and CO_2_ concentration [64]. These environmental factors regulating morphogenesis are controlled via multiple signalling pathways, depending on the physiological cues present. Pathways such as the cAMP-PKA and Ras1-MAPK are both initiated by the guanine nucleotide binding protein Ras1, but diverge their signalling cascades downstream of Ras1 activation. Most components of these pathways are critical for filamentous growth, including the downstream transcriptional regulator Efg1 [39]. Several additional MAPK pathways are also critical regulators of *C. albicans* morphogenesis; the protein kinase C (PKC) and the high osmolarity glycerol (HOG) MAPK pathways are involved in maintaining cell wall integrity and have components that modulate morphogenesis [68,69,70]. Other pathways such as the pH, TOR, and cell cycle arrest pathways all control filamentous growth and the expression of hyphae-specific genes via different signalling cascades [39]. Additionally, the heat shock factor Hsp90 governs temperature-dependent morphogenesis in *C. albicans* via the cAMP-PKA pathway [71,72,73], and the heat shock transcription factor Hsf1 also regulates filamentous growth by compromising the Hsp90 function [74]. 

Hyphal formation in *C. albicans* can also be negatively regulated by different signalling cues, and, most notably, by the fungal-produced quorum sensing molecule farnesol [75,76,77]. Farnesol initiates repression of filamentous growth by targeting key signaling cascades involved in the yeast-to-hyphal growth transition [78]. Farnesol targets the cAMP-PKA pathway via hydrophobically disrupting the membrane-bound Ras1-Cyr1 complex that affects downstream cAMP production [79]. In addition to the cAMP-PKA pathway, farnesol acts through Cup9 and Ubr1 signaling. When *C. albicans* cells are released from farnesol inhibition, the ubiquitin protein ligase Ubr1 degrades the transcriptional repressor Cup9, which in turn de-represses expression of *SOK1*, which is required for degradation of Nrg1, and initiation of filamentous growth [80,81,82]. Farnesol has also been shown to regulate multiple kinases and transcription factors responsible for hyphal formation [83], establishing this molecule as an important modulator of the yeast to hyphal transition. For *C. albicans*, farnesol is only produced under aerobic growth conditions [84].

Ultimately, activation of filamentous growth pathways and transcription factors reads the expression of ‘hyphal-specific genes’, which are specifically induced or upregulating in filamentous growth forms. These include a number of cell surface adhesin proteins that aid in fungal adhesion, including agglutinin-like sequence (*ALS*) family proteins, and ‘hyphal wall protein’ *HWP1* [85]. Other genes that are upregulated upon yeast-to-filamentous growth transition in *C. albicans* include secreted aspartic proteases, encoded by *SAP* genes [86,87] proteins that exhibit extracellular proteolytic activity, aid in the degradation of host proteins, and are tightly linked to fungal virulence [88,89]. *ECE1* is also a hyphal-specific gene in *C. albicans*; the proteolytic cleavage of the Ece1 protein results in production of the peptide toxin candidalysin [59]. Other hyphal-specific genes associated with the filamentous form of *C. albicans* include *HGC1* [90], *DDR48, RBT1,* and *RBT4* [91,92].

Transcriptional control over genes governing morphogenesis is critical, and multiple transcription factors are core modulators of the different *C. albicans* morphogenetic circuitry. For example, the filament-specific transcriptional regulator *UME6* is needed for proper expression of these hyphal-specific genes [93]. In experiments where Ume6 is constitutively expressed, it forms hyphae even in non-filament-inducing cues [94]. *EFG1* is also a major transcriptional regulator of filamentation that governs yeast to hyphal morphogenesis downstream of cAMP-PKA signaling [95,96,97]. Many other transcription factors such as *FLO8*, *CPH1, EED1, CZF1* and *TEC1* are also positive regulators of filamentous growth [98,99,100,101]. Other transcription factors such as *TUP1, NRG1 and RFG1* are negative regulators of filamentation. They act by repressing hyphae specific genes and when null mutants are created, hyphal formation occurs in *C. albicans* without the need for any inducing cues [102,103,104]. Together, these factors are uniquely expressed or upregulated in *C. albicans* filamentous cells, and are responsible for the phenotypes associated with filamentous growth.

### 2.2. Biofilm Formation

*Candida* spp., are frequently found as part of microbial biofilms in both their yeast and filamentous growth states. Biofilms are structured microbial communities that are comprised of single or multiple microbial species, encased in an extracellular matrix (ECM). Similar to other microbial biofilms, *Candida* biofilms tend to be more resistant to host defences and antimicrobial agents, particularly azoles, and can be an important component of persistence within the host [12,29,105,106]. *Candida* biofilms are composed of multiple layers of microbial cells and a matrix of extracellular material that may vary between species, consisting of polysaccharides, including β-1,3-glucan, β-1,6-glucan, mannans, and proteins [107,108]. Biofilm associated *C. albicans* cells contain cell walls which are twice as thick, and contain more carbohydrates and β-1,3-glucan, than their planktonic cell counterpart [107]. While *C. albicans* is commonly associated with biofilm growth, many other non-*albicans Candida* species are also capable of biofilm growth, as well as filamentous growth, such as *C. tropicalis, C. parapsilosis,* and *C. auris* [37,109]. Biofilms significantly increase fungal pathogenicity due to their ability to secrete a protective ECM, and anchor robustly to host tissues [12]. 

The ability of several *Candida* species, including *C. albicans*, *C. tropicalis,* and *C. parapsilosis* to transition between distinct morphological states, and the large number of surface adhesins associated specifically with the hyphal form, such as *ALS3* and *HWP1*, both play key roles in maintaining biofilm formation [37,110,111]. In many cases, *C. albicans* mutants unable to undergo morphogenesis are also defective in forming biofilms [112]. Filamentous cells contribute structural integrity and strength to a biofilm and act as a support for adherence of yeast cells [38], while yeast cells may allow for dissemination of biofilm cells within a host [110]. Filamentous cells also enable *C. albicans* to invade host cells and tissues, and allow biofilms to mature [12]. The primary morphological composition of the biofilm dictates its strength: biofilms that are composed of 50% or more hyphae are more robust and more difficult to disrupt [64,112], due to filamentous cells expressing more adhesin proteins, facilitating growth of more mature, robust biofilms [38]. Biofilm formation is also regulated by a complex transcriptional network that is intrinsically linked to morphogenetic switching in polymorphic *Candida* spp. [111]. This network is governed by several key transcription factors, including Ndt80, Rob1, Bcr1, Efg1, Tec1, Brg1, Flo8, Gal4, and Rfx2, many of which are also crucial for filamentous growth [113,114].

## 3. Fungal Treatment: Classes of Antifungals and Drug Resistance

### 3.1. Antifungal Drugs 

Fungal morphogenesis has links with treatment and response to antifungal drugs. Here, we briefly review the major classes of antifungal drugs and mechanisms of resistance. Due to the relatively close evolutionary relationship between eukaryotic fungal pathogens and mammalian cells, there are limited cellular targets which can be exploited for use in antifungal drug therapy, while also exhibiting limited toxicity against human patients. The four major classes of antifungal drugs used for treatment of *Candida* infections are the azoles, polyenes, echinocandins, and flucytosine.

Azoles target production of ergosterol [115]: a key sterol which serves as the functional equivalent of cholesterol within mammalian cells, in order to regulate the integrity and fluidity of the fungal cell membrane [116]. Azoles exert their fungistatic effect on *Candida* by targeting ergosterol production. They function by specifically targeting the enzyme lanosterol 14α-demethylase, (that is encoded by the *ERG11* gene in *Candida*) which is a key enzyme in the ergosterol biosynthetic pathway [117]. Azoles contain a free nitrogen atom which binds to the heme group at the active site of Erg11, preventing the demethylation of lanosterol [118,119,120] and effectively blocking ergosterol production. 

The echinocandins are a class of antifungal drugs that, unlike azoles, typically act in a fungicidal manner against most *Candida* species [121]. Echinocandins exhibit low toxicity against the host and target the fungal cell wall specifically by acting as non-competitive inhibitors of the enzyme (1,3)-β-d-glucan synthase [121]. This inhibition results in the disruption of the synthesis of the carbohydrate β-1,3-glucan, a major component of the fungal cell wall [122]. Disruption of (1,3)-β-d-glucan synthase causes severe stress on the fungal cell wall and results in loss of cell wall integrity, followed by lysis [123]. 

Polyenes are potent, amphipathic compounds, which contain both hydrophobic and hydrophilic regions, giving these drugs a unique molecular structure capable of directly targeting and strongly binding to ergosterol in the fungal cell membrane [124]. Similar to echinocandins, they exert a fungicidal effect on *C. albicans*, as the binding of a polyene to ergosterol, forms a large drug-lipid complex which spans the fungal cell membrane. This forms a leaky channel, disrupting the membrane and allowing the escape of cellular ions out of the cell which significantly disrupts the proton gradient of the cell [125]. Recently, the production of reactive oxygen species (ROS) has also been proposed to play a role in the fungicidal activity of the polyene amphotericin B [126,127]. Amphotericin B has an impact on fungal redox homeostasis [127], and alters *C. albicans* cellular metabolism and respiration, ultimately leading to the production of intracellular ROS, which is lethal to the cell [126]. Polyenes have been well known antifungals for over 50 years, but their clinical use has been limited due to both poor solubility and strong nephrotoxicity, caused by structural similarities between ergosterol, and its mammalian analogue cholesterol [128,129]. 

Finally, the pyrimidine analog flucytosine works by blocking both RNA and DNA biosynthesis pathways in fungi. Flucytosine is rarely, if ever, used in antifungal monotherapy against *Candida* infections, due to the rapid and frequent development of resistance to this antifungal [130]. Typically, flucytosine is used in combination with other antifungal, such as amphotericin B. Flucytosine (in combination with amphotericin B), is recommended for the treatment of drug-resistant *Candida* infections, including fluconazole-resistant *C. glabrata* infections [131].

### 3.2. Antifungal Drug Resistance

*Candida* species are able to evolve various drug resistance against all three classes of antifungals, and some have intrinsic resistance to antifungals (Table 1). Resistance to the azoles are most dominant and widely reported in literature, due to their extensive clinical use over the past few decades [129]. Four major mechanisms have been reported to induce azole resistance in *Candida*, with many of these mechanisms being reported in multiple *Candida* species, and some azole-resistant isolates demonstrating multiple resistance strategies [132]: 1. Point mutations in the gene *ERG11* at different “hotspot” regions which confers poor or insufficient azole binding [117,133,134,135,136,137]; 2. Transcriptional upregulation of the gene *ERG11* resulting in upregulated ergosterol production [138,139], or amplification of the *ERG11* gene [140,141]. Evidence has also suggested that in *Candida* species, *ERG11* may be upregulated by the zinc-cluster transcriptional regulator Upc2p [142,143,144,145]; 3. Overexpression or amplification of multiple classes of drug efflux pumps, including the ABC transporters *CDR1* and *CDR2* [117,146,147,148] and the major facilitator superfamilies (MFS) proteins, such as transporter genes *MDR1* [149,150,151], which may be overexpressed in azole-resistant *Candida* isolates (*C. krusei*, is intrinsically resistant to azoles, which is associated with upregulation of efflux pumps [117]); 4. Regulation and induction of numerous cellular stress response pathways such as the phosphatase protein calcineurin, and the molecular chaperone Hsp90 [39,123]. 

Echinocandin resistance has been linked to mutations occurring within *FKS* genes, including the essential gene *FKS1* [152] and its paralog *FKS2* [153]. These *FKS* genes encode for the catalytic subunit (1,3)-β-D-glucan synthase, which is the target of echinocandin binding [121]. *FKS* mutations confer a structural alteration which results in poor echinocandin binding [152] and have been reported to occur within two “hotspot regions” within *FKS1*, the first of which mutates Ser645 to either Tyr645, Pro645, or Phe645; the second hotspot region has been mapped to point mutations occuring between the amino acids 1345 to 365 [154]. In *C. albicans*, mutations within *FKS1* alone are sufficient enough to confer echinocandin resistance. However, within some other *Candida* species, such as *C. glabrata*, mutations within both *FKS1* and *FKS2* have been linked to echinocandin resistance [153]. Echinocandin resistance has also been linked to stress response pathways, including elevated expression levels of the genes *CNB1* and *MID1* [154], which play a role in the calcineurin cellular stress response signalling pathway [123]. The calcineurin, PKC and HOG pathways have all also been implicated in regulating the increased expression of chitin synthase genes in response to cell wall stress due to echinocandins [39]. 

Antifungal resistance to polyenes is rare, but has been reported in diverse *Candida* species, due primarily to alterations in membrane sterol composition [155,156,157,158,159]. In *C. albicans*, resistance has been reported due to mutations in the C-5,6-desaturase enzyme, which blocks ergosterol biosynthesis and leads to the accumulation of an alternative sterol in the fungal cell membrane [160]. Some *Candida* species, such as *C. lusitaniae* and *Candida guilliermondii* have been reported to demonstrate intrinsic resistance to the polyene amphotericin B [39], which may occur due to mutations in sterol biosynthesis pathways or other components of their cell membrane [128]. 

## 4. *Candida* Morphogenesis, Antifungal Drugs and Drug Resistance

In this section, we highlight the connections between cellular morphogenesis in *Candida* species, and treatment with antifungal agents. In particular, we analyze how existing antifungal agents are able to modulate cellular morphogenesis; the conserved signaling pathways involved in morphogenesis and the response to antifungal drugs, including antifungal drug resistance pathways; and how *Candida* biofilms connect fungal morphology to resistance to antifungal drug treatment. Finally, we will discuss novel ‘anti-virulence’ drugs, that specifically target fungal morphogenesis as a means to limit the pathogenicity of *Candida* infections, and may serve as innovative new strategies to treat fungal infections.

### 4.1. Effects of Antifungals on Fungal Morphology

**Figure 1:**
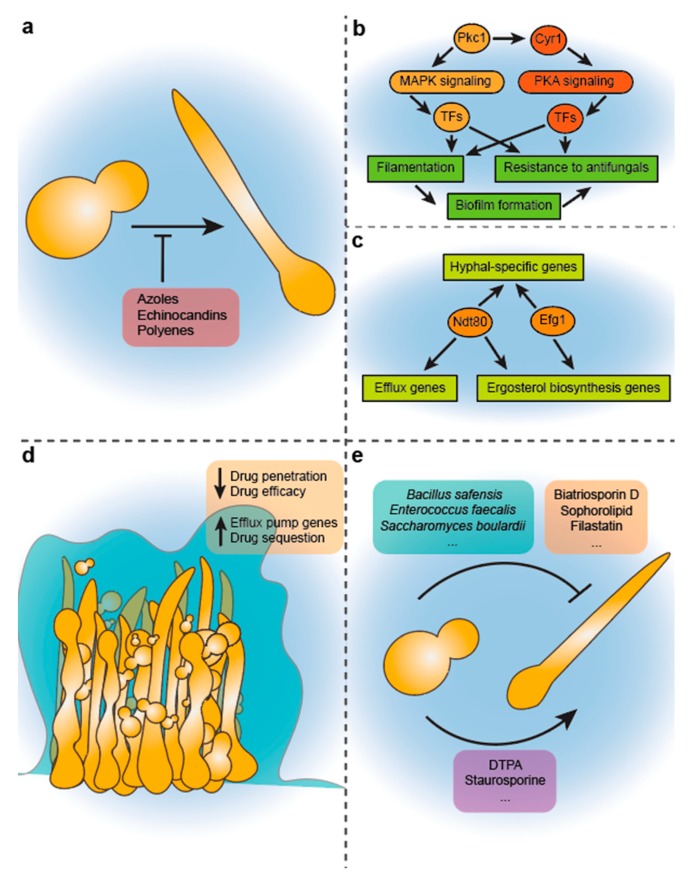
Linking morphogenesis with antifungal drug treatment and drug resistance in *Candida*. (**a**) antifungals and their impact on morphogenesis. *Candida* morphogenesis can be blocked with treatment with antifungal compounds, including azoles, echinocandins, and polyenes [161,162,165,166,167]. This morphogenetic inhibition is seen even at sub-inhibitory concentrations of all three classes. (**b**) interactions between signalling pathways that govern morphogenesis and drug resistance. The protein kinase C (PKC)-MAPK pathway and the cAMP-PKA pathway are both involved in drug resistance and morphogenesis in *Candida*. Depicted are the downstream effects of PKC which regulates MAPK signalling to maintain cell wall integrity when exposed to drugs. Pkc1 has been found to regulate Cyr1 [171], which is a part of the cAMP-PKA signalling pathway, which governs filamentous growth [39]. Pkc1 is implicated in regulation of both drug resistance [172] and hyphal formation [171]. Signaling from the PKA pathway also plays a role in drug resistance via Cyr1 signaling [173,174], as well as morphogenesis [39]. Downstream transcriptional regulators of both MAPK and PKA pathways ultimately regulate morphogenesis and resistance to antifungals. Morphogenesis and filamentous growth also contribute to biofilm formation, which in turn enhances antifungal resistance. (**c**) transcriptional regulation of antifungal drug resistance and morphogenesis. Transcription factors such as Efg1 and Ndt80 have previously been linked to modulating expression of hyphal specific genes and promoting filamentation [95,96,97,175,176]. Both of these transcriptional regulators are also involved in drug resistance, via regulation of genes involved in drug efflux and/or ergosterol biosynthesis [176,177,178,179,180]; (**d**) coupling *Candida* morphogenesis and drug resistance through biofilm formation. Biofilms of polymorphic *Candida* species are typically composed of diverse cellular morphologies: hyphae, pseudohyphae and yeast [38]. Once established, these polymorphic biofilms secrete an extracellular matrix (ECM) that helps shield *Candida* from the external environment. Biofilms play a significant role in antifungal drug resistance by preventing antifungal penetration, sequestering antifungals, upregulating antifungal drug efflux, and metabolic regulation that limits antifungal activity [105,181,182,183]; (**e**) novel compounds for targeting *Candida* morphogenesis. New compounds are being discovered that can modulate filamentation in *Candida* species, and may serve as novel therapeutic strategies for treating fungal infections. Examples include drugs (i.e., filastatin), and compounds secreted by competing microbes (i.e., *Bacillus safensis*) that prevent filamentation, and drugs that enhances filamentation (i.e., diethylenetriaminepentaacetic acid (DTPA)).

The interactions between fungal morphogenesis and exposure to antifungal agents have been previously studied amongst *Candida* species upon treatment with antifungal drugs. It has been well documented that the azoles—one of the most widespread antifungals—also have a direct effect on *C. albicans* morphology, and prevent the formation of hyphae (Figure 1a) [161,162]. The effect of azole-mediated hyphal repression is prominent enough that at subinhibitory azole concentrations hyphal branching is restricted, and at clinically relevant doses, the yeast-to-hyphal transition is prevented entirely [161,162]. It was shown that *C. albicans* cells treated with azoles produced higher levels of the farnesol [163], a fungal-produced quorum sensing molecule with the ability to block filamentous growth in *C. albicans*. This occurs because azoles target enzymes involved in sterol biosynthesis, and azole treatment leads to a build up of the sterol biosynthetic intermediate, farnesyl pyrophosphate, which indirectly stimulates the over-production of farnesol [163,164]. As previously described, farnesol is able to inhibit filamentous growth in *C. albicans* by targeting key pathways, such as the cAMP-PKA cascade and via Ubr1-mediated degradation of Cup9 [80], which are involved in the yeast-to-filamentous growth transition [78]. Under anaerobic growth conditions, *C. albicans* does not produce farnesol, and is more resistant to antifungals, including azoles and amphotericin B, potentially due to reduced carbon flow through the sterol pathway [84], further highlighting the connection between quorum sensing and antifungal resistance. Together this explains a critical connection between a clinically-relevant class of antifungal agents (the azoles), and the repression of morphogenesis—a critical virulence trait in *Candida* pathogens. This may also explain the success of the azoles as therapeutics.

Aside from azoles, polyenes and an echinocandin-like antifungal have also been documented to disrupt the yeast-to-hyphal morphogenetic transition in *C. albicans* (Figure 1a) [165,166,167]. For polyenes, it has been demonstrated that sub-MIC concentrations of amphotericin B can limit the transition between yeast and filamentous growth [165,167]. Similarly, the echinocandin-like antifungal mulundocandin prevents yeast to filamentous growth when administered at sub-MIC levels [165]. In the case of treatment with echinocandins, there are many changes in cellular and cell wall composition that occur in *Candida* species treated with these antifungals, including increase in cell wall chitin content, decrease in β-glucans, and the upregulation of cell surface proteins [168]. The prevention of hyphal formation by the echinocandin-like drug mulundocandin may be due to these alterations in cell wall composition, resulting in disrupted cellular morphogenesis and cell elongation. 

### 4.2. Fungal Morphogenesis of Drug Resistant Isolates

Aside from antifungal drugs that are able to modulate cellular morphogenesis in *C. albicans*, there is also a relationship between morphogenesis and resistance to antifungal drugs. It has been observed that there is a correlation between resistance to antifungals and the ability to undergo filamentous growth [161,169]. For azoles, when *C. albicans* clinical isolates with variable levels of resistance to azole antifungals were grown under filament-inducing conditions, and, in the presence of an azole drug, drug-resistant isolates were able to form hyphae more efficiently than susceptible isolates [161,170]. In *C. lusitaniae*, there is also an association between resistance to the polyene amphotericin B, and pseudohyphal filamentation: phenotypic switching produces a distinct phenotype (dark brown colony pigmentation on copper sulfate media), which is associated with both pseudohyphal growth as well as an slightly elevated MIC relative to the dominant “light brown” phenotype [50]. Similarly, for echinocandin-resistant *C. albicans* strains, there is a correlation between resistance and cellular morphogenesis: strains harbouring an *fks1* mutation, rendering cells resistant to echinocandins, have defects in filamentous growth [169]. Interestingly, *fks1* mutants with impaired filamentation are those with the highest relative levels of cellular chitin in the resistant strains [169], suggesting that echinocandin resistance-mediated over-production of chitin and altered cell-wall composition limits filamentation. 

### 4.3. Shared Cellular Signaling Pathways Mediating Candida Morphogenesis and Drug Resistance

Important links have been discovered between the cellular signalling pathways that modulate morphogenesis in *Candida*, and those that regulate resistance to antifungal drugs. Indeed, many of the central morphogenetic pathways, such as the cAMP-PKA and MAPK pathways, are additionally involved in antifungal drug resistance (Figure 1b) [39]. For instance, in *C. albicans*, mutation or deletion of Cyr1 (the adenylate cyclase that synthesizes cAMP) renders cells unable to filament, and hypersensitive to azole antifungals [173,174]. This antifungal sensitivity is conferred, at least in part, due to lack of upregulation of the drug efflux pump Cdr1 in Cyr1-deficient *Candida* strains [174]. Pkc1, a component of PKC-MAPK signaling in *C. albicans* is an important regulator of antifungal drug resistance [172]. Recent studies have also discovered functions in the PKC pathway that also regulates filamentation: the protein kinase Pkc1 can function alongside the cAMP-PKA pathway to stimulate activation of Cyr1 to generate cAMP, and induce filamentation [171]. Since Pkc1 is known to interact with actin in *S. cerevisiae* [184], and Cyr1 also interacts with G-actin [185], it may be that actin is required for Pkc1 to govern morphogenesis in *Candida*. Other MAPK-related response regulators have similarly been implicated in filamentous growth and drug sensitivity in *C. lusitaniae* [186].

The essential molecular chaperone Hsp90 is a central regulator of fungal morphogenesis and antifungal drug resistance. Hsp90 is involved in temperature-dependent morphogenesis in *C. albicans*, such that inhibition of Hsp90 leads to enhanced filamentous growth, via the cAMP-PKA signaling pathway [72]. Inhibition of Hsp90 also blocks the emergence of resistance to the azoles, enhances antifungal sensitivity in azole-resistant strains [187], and reduces tolerance and resistance to echinocandins [123]. Other proteins and pathways involved with Hsp90 signalling are similarly involved in both morphogenesis and antifungal drug resistance, including the Hsp90 client protein and stress response factor calcineurin [123,188,189,190], and the Hsp90 co-chaperone Sgt1 [71]. Hsp90 plays a conserved role in mediating morphogenesis and azole tolerance in *C. auris* [53]. Additionally, lysine deacetylases (KDACs), including Hos2, Hda1, Rpd3, and Rpd31, are key regulators of both antifungal drug resistance and morphogenesis in *C. albicans* [191,192], at least in part via their deacetylation and regulation of Hsp90 function [191,192]. The ability of KDACs to mediate drug resistance via Hsp90 is conserved across *C. tropicalis* and *C. guilliermondii* [192].

Additional cellular factors have a shared role in the modulation of fungal morphogenesis and susceptibility to antifungal agents. There are several examples of *Candida* mutant strains that are both defective in filamentous growth, and demonstrate increased susceptibility to antifungal agents. For instance, the *C. albicans PMT* genes encode a family of protein *O*-mannosyltransferases that are critical for *O*-mannosylation of fungal secretory proteins, and are involved in both morphogenesis and antifungal drug resistance. Genetic deletion or depletion of *C. albicans PMT* genes, including *PMT1*, *PMT2*, *PMT4*, and *PMT6* inhibits hyphal formation under diverse environmental growth conditions, and renders cells highly susceptible to cell wall and cell membrane stressors, including the azole antifungal ketoconazole [193,194]. Disruption of the sphingolipid biosynthetic gene *IPT1* also renders cells sensitive to azole antifungals, and defective in hyphal formation [195]. This effect is has also been observed with the sphingolipid genes *FEN1* and *FEN12*, although with the polyene amphotericin B instead of azoles [196]. This suggests that several key factors involved in either cell wall modelling or membrane composition, which in turn affects the capacity of the cell to undergo cellular morphogenesis and respond to the presence of antifungal agents. 

Factors involved in ergosterol biosynthesis and function are also involved in mediating antifungal drug resistance and morphogenesis. For instance, the squalene epoxidase *ERG1*, which catalyzes an essential step in the biosynthesis of ergosterol, is involved in resistance to azoles and hyphal formation [197]. *C. albicans* mutants depleted of *ERG1* show abnormal sterol composition, leading to increased sensitivity to both azole and polyene antifungals, along with an inability to form hyphal cells [197]. Similarly, the *C. albicans O*-acyltransferase *GUP1* gene is also involved in mediating drug resistance to the azoles via alterations in membrane ergosterol constitution and distribution, and is strongly implicated in hyphal growth and biofilm formation [198]. 

#### Transcription Factors Coupling Morphogenesis and Antifungal Drug Susceptibility

There are also many transcription factors that are recently being understood to control circuitry involved in both *Candida* morphogenetic programs, as well as antifungal drug resistance (Figure 1c). For instance, in *C. albicans*, the Zn cluster transcription factors Tac1 and Znc1 are upregulated by farnesol (which blocks *C. albicans* filamentation), and in turn bind to the promoter of efflux pump genes such as *CDR1* and *CDR2*, leading to their overexpression [199]. This suggests that drug treatment with the azoles, which, as previously described, leads to increased farnesol production [163], limiting filamentous growth, and in turn activating Tac1 and Znc1, can further lead to increase drug efflux and thus resistance. The well-characterized morphogenesis transcription factor Efg1, is also linked to susceptibility to azoles, polyenes and echinocandins in different *Candida* species (Figure 1c) [177,178]. *C. albicans efg1∆* mutant strains downregulate the expression of *ERG11,* and have decreased levels of ergosterol, resulting in enhanced susceptibility to azoles and polyenes [200]. Other studies have found that in *C. parapsilosis*, *efg1Δ* mutants were also susceptible to echinocandins [178]. 

The transcription factor Ndt80 has also been implicated in resistance to antifungals and morphogenesis in *C. albicans* (Figure 1c). Ndt80 regulates drug resistance by upregulating expression of the efflux pumps *CDR1, CDR2,* and *MDR1* [176,179] as well as ergosterol biosynthesis genes, such as *ERG3* and *ERG11* [180]. Ndt80 also plays an essential role in filamentous growth, as this transcriptional regulator interacts with hyphal-specific gene promoters, and upregulation of factors such as *HWP1, ECE1, RBT4,* and *ALS3* [176], and deletion of *NDT80* renders *C. albicans* unable to filament [175].

The transcription factor *CAS5* and the coactivator, *ADA2* also play a key role in the response to antifungal drugs, and cell morphology. Deletion of these transcription factors renders *C. albicans* hypersensitive to caspofungin, and both factors are required for expression of numerous caspofungin-responsive genes [201]. Similarly, disruption or deletion of both Ada2 and Cas5 renders *C. albicans* hypersensitive to fluconazole and enhances azole-mediated killing, via regulation of efflux pumps and ergosterol biosynthesis genes [151,202]. Ada2 and Cas5 are also both important for *C. albicans* filamentation in vivo, and contribute to fungal virulence [203,204]. Recently, a new role for Cas5 was uncovered in *C. albicans*, as a key regulator coupling the response to cellular stress, drug resistance, and cell cycle regulation [205], which may help explain the pleiotropic role of this transcription factor.

It is likely that many additional transcriptional regulators are similarly involved in morphogenesis and antifungal susceptibility. For example, Ume6 is a transcription factor that is critical for *C. albicans* morphogenesis [94,206,207]. Given that Ume6 interacts directly with the cell wall biogenesis protein Sun41 [208], which itself has been implicated in basal caspofungin [209] and fluconazole tolerance [210], it may play a pleiotropic role in morphogenesis as well as susceptibility to antifungal agents. Future research will likely identify additional signaling networks and transcriptional regulators with key functions in coupling antifungal susceptibility with cellular morphogenesis in *C. albicans*.

### 4.4. Biofilm Formation and Drug Resistance

One of the key connections between *Candida* morphogenesis and antifungal drug resistance, is the formation of biofilms (Figure 1d). As previously described, amongst polymorphic *Candida* species, biofilms are typically comprised of a dense community of yeast and filamentous cell types [37,38,211]. These polymorphic *Candida* biofilm communities also play a significant role in resistance to antifungal drugs, and confer enhanced drug resistance [12,105,183]. Biofilms provide many mechanisms for drug resistance, including prevention of antifungal penetration, upregulation of antifungal drug efflux pumps, drug sequestration, and the presence of persister cells (Figure 1d) [105,181,182,183]. 

Biofilms prevent antifungals from penetrating cells due to the chemical composition of the thick extracellular matrix (ECM), allowing cells to grow and propagate within a biofilm without interference [105]. Matrix polymers in the ECM such as polysaccharides, including β-1,3-glucan, β-1,6-glucan, mannans, and proteins, are thought to confer antifungal resistance to biofilms as they prevent deep penetration of antifungals into the biofilm [105,107]. It was recently found that in *C. albicans*, biofilms produce extracellular vesicles (EVs) that play a central role in biofilm matrix production and resistance to antifungal drugs [210]. EVs, which are membranous compartments released by almost all cells, are commonly found amongst fungi, and carry proteins, lipids, polysaccharides, and RNA [212,213,214]. In *C. albicans*, EVs produced by planktonic cells versus those in biofilms, have a unique proteomic composition; biofilm-associated EV composition is very similar to the protein and polysaccharide composition of the ECMs, including a significant amount of mannan and glucan [210]. It has recently been shown that *C. albicans* mutants defective in the ESCRT (endosomal sorting complexes required for transport) pathway, have decreased vesicle production and are hypersusceptible to fluconazole during biofilm growth [210], likely due to decreased vesicle delivery of ECM materials (i.e., mannan and glucan), and thus decreased ECM-associated drug sequestration. This novel finding reinforces the critical link between fungal biofilm ECM and antifungal drug resistance, and suggests ESCRT proteins as putative targets to limit drug resistance in *C. albicans* biofilms.

If drugs bypass this defense and enter the biofilm-associated cells, upregulated efflux pumps force antifungal drugs out of the cells, to prevent any further damage to *Candida* [215]. Studies have shown that while efflux pumps have minimal effects on mature biofilms, during the early phases of biofilm formation, efflux pumps play a significant role in antifungal resistance and are significantly upregulated in early biofilms [105]. Drugs that were not pumped out by efflux pumps and remain in the cell undergo drug sequestration in which the drugs are compartmentalized in the cell in less harmful locations [105]. Drug sequestration of azoles, echinocandins, pyrimidines, and polyenes is regulated by β-1,3 glucan, a major carbohydrate component of *Candida* biofilms [105]. 

Persister cells are a type of metabolically dormant cell that may arise in biofilms, and which may be more tolerant to antifungal drug treatment. These persister cells may be involved in antifungal tolerance and recalcitrant infections [181]. These metabolically dormant *Candida* cells are found in biofilms [216,217], and this allows these biofilms to be more tolerant to antifungal drugs, compared to planktonic fungal cells [181,216,217,218]. Typically, fungicidal drugs induce cell death via the production of reactive oxygen species (ROS) [126]; however, persister cells are capable of dealing with larger quantities of ROS, and survive via activation of an oxidative stress response [181]. This response involves an overexpression of superoxide dismutases (SODs), which assist in protecting cells from ROS and allows biofilm-associated cells to survive in the presence of antifungal drugs [181]. 

While *Candida* biofilms are, in general, more resistant to antifungals, they are particularly resistant to the azole class of antifungals [106], and are generally more susceptible to the echinocandins and liposomal formulations of the polyene amphotericin B [219,220]. This may be due to several factors, including the fact that *Candida* biofilms have an altered sterol composition relative to planktonic cells [221], which may disproportionately affect resistance to the sterol biosynthesis-targeting azoles. Biofilm cells also overexpress the target of the azoles, *ERG11* [222], which may promote azole-specific resistance. Additionally, *Candida* cells in biofilms overexpress drug-efflux pumps [106]; while this may increase overall resistance to antifungals, resistance to azoles is uniquely affected by the overexpression of the MFS transporter *MDR1* in biofilms [223,224,225,226].

### 4.5. New Antivirulence Compounds That Target Candida Morphogenesis

We have established that there is a connection between antifungal drugs and *Candida* morphogenesis: existing antifungal agents can modulate filamentation (and thus virulence), and many signaling pathways are shared between those involved in cellular morphology, and those involved in the response and resistance to antifungal agents. Here, we focus on how anti-morphogenesis compounds can be harnessed as novel therapeutics for the treatment of fungal pathogens (Table 2).

Given the limited number of clinically-available antifungal drugs, increasing rates of antifungal drug resistance, and emergence of multi-drug resistant pathogens, such as *C. auris* [32], there is a renewed urgency for the development of new strategies to tackle fungal pathogens. As described earlier, existing antifungal drugs create a very strong selection pressure, and *Candida* species are able to evolve resistance to antifungal drugs via multiple mechanisms [227]. Recently, there has been a research emphasis on identifying and characterizing drugs that target virulence processes, such as fungal morphogenesis, that may limit or inhibit pathogenicity of a microbial organism, while still allowing it to live (Figure 1e) [228,229,230,231]. It is suggested that such anti-virulence compounds will create a weaker selection pressure on the microbes, thus preventing selection for the rapid evolution of drug resistance. Furthermore, by allowing survival of their microbial targets, anti-virulence compounds would theoretically be less disruptive to the ecological balance of the host microbiome.

In the case of *C. albicans*, the inhibition of morphogenesis is an excellent target for anti-virulence drugs (Table 2), given that cellular morphogenesis is coupled with fungal virulence and inhibiting the process may aid in better containment and clearance by the host’s own immune system [78,232]. As a result, several screens have been conducted to identify new small-molecule inhibitors that target the morphogenetic transition in *Candida* pathogens [78,233,234,235,236,237,238]. The majority of these experiments have focused on treatment of *Candida* strains with the small molecules under conditions which promote filamentous growth, followed by either a physical or genetic assessment of hyphal formation. Here, we will review some key findings of novel, anti-morphogenesis inhibitors in *Candida* pathogens (Figure 1e), with potential applications as innovative therapeutic strategies for limiting the virulence of these pathogens.

Zhang et al. recently discovered that *C. albicans* treated with the small phenolic compound Biatriosporin D prevents yeast-to-hyphal morphogenesis [239]. It does so by downregulating the expression of the hyphal-specific genes *ALS3, HWP1,* and *ECE1* by directly affecting the cAMP-PKA pathway via the regulation of the yeast’s intracellular cAMP levels. Cells treated with Biatriosporin D showed a dose-dependent decrease in intracellular cAMP levels and exogenous cAMP can partially restore hyphal formation. Biatriosporin D was also found to upregulate the expression of Dpp3, which is involved in the synthesis of the hyphal-inhibiting quorum-sensing molecule farnesol [239,240]. The combination of increased farnesol production, decreased cAMP levels, and low toxicity, renders Biatriosporin D a strong inhibitor of filamentation, and an emerging therapeutic candidate. 

Some compounds, such as the glycolipid sophorolipid, has previously shown antimicrobial activity [248]. Haque et al. have investigated the antifungal effects on planktonic *Candida* spp. and also demonstrated that sophorolipid prevents hyphal formation [241]. Upon treatment with sophorolipid, biofilm formation was significantly disrupted and the expression of hyphal-specific genes (*HWP1, ALS1, ALS3, ECE1* and *SAP4*), were downregulated [241]. This downregulation of hyphal genes abrogated the capacity of *C. albicans* to form biofilms, as well as increased the efficacy of treatment with other antifungals, implying its potential utility in combination with antifungal treatment. 

Recently, Romo et al. identified a novel group of bioactive compounds with a common biaryl amide core structure that were potent inhibitors of filamentation in *C. albicans*. Genetic analysis suggests that these inhibitors function downstream of multiple signalling pathways and are likely impacting filamentation via the transcription factor Brg1. After confirming the ability of these compounds to inhibit filamentation and biofilm growth in vitro, this group performed in vivo studies using mouse models of disseminated and oropharyngeal candidiasis. The hyphal-inhibiting drugs were able to increase mouse survival, and inhibit filamentation in vivo, as demonstrated by lack of hyphal cells in histological samples of kidneys from infected mice [237]. Importantly, while these drugs blocked hyphal growth, they did not impact fungal survival, as overall fungal burdens remained similar between treated and untreated mice [237].

Another potent inhibitor of *Candida* filamentation is the compound filastatin, which blocks the yeast-to-hyphal morphological transition in *C. albicans*, and also inhibits cellular adhesion, biofilm formation, and pathogenesis [238]. Filastatin inhibits filamentation in diverse environmental conditions, in both liquid and solid growth media [238]. Genetic pathway analysis was performed to identify the mechanism of action of this inhibitor, and revealed that it likely acts downstream of two key transcriptional regulators: Efg1 and Cph1 [238]. These transcription factors are the terminal transcriptional regulators of the cAMP-PKA pathway and MAPK pathway, respectively, and both are key regulators of filamentous growth in *C. albicans*. Another recent report used similar chemical-genetic profiling to identify the function of hyphal inhibitors Niclosamide and TCSA (Tri-Chloro-Salicyanilide) [233]. These small molecule inhibitors, which block hyphal formation in *C. albicans* and inhibit biofilm growth of *C. albicans* and *C. auris*, were similarly found to act on effectors downstream of cAMP-PKA and MAPK signalling pathways [233].

In addition to small molecule inhibitors of filamentation identified through drug screening assays, another relevant reservoir for anti-morphogenesis compounds is other microorganisms which may coexist and compete with *Candida* species (Figure 1e). Previous work has shown that bacteria such as *Bacillus safensis* can produce a chitinase compound with antipathogenic capabilities which can specifically target *C. albicans*’ capacity to form functional hyphae [249]. *B. safensis* is able to directly bind to *C. albicans* and secrete a chitinase that can destabilize the cell wall and prevent proper hyphal formation. This leads to the antivirulent effect of halting *C. albicans’* morphological transition from yeast to hyphal growth, and also efficiently preventing biofilm formation. Similarly, *Enterococcus faecalis* bacteria produce the EntV bacteriocin, which is able to inhibit hyphal morphogenesis, biofilm formation, and virulence in *C. albicans* [243]. Many *Lactobacillus* species are able to inhibit hyphal formation of *C. albicans* through the production of organic acids that lower the pH of the surrounding microenvironment, promoting yeast-form growth [250], and *Escherichia coli* can modulate biofilm formation and hyphal growth of diverse *Candida* pathogens [251]. *Candida* species can also regulate hyphal formation of other competing *Candida* organisms: *C. krusei* and *C. glabrata* can prevent hyphal formation in *C. albicans*, resulting in downregulation of *HWP1* expression [252]. The yeast species *Saccharomyces boulardii* shows similar anti-virulence effects on *C. albicans*, and can block the yeast to hyphal growth transition via the secretion of capric acid [244,253]. Identifying additional factors secreted by competing microorganisms may provide a practical method to identify new drugs to inhibit *Candida* morphogenesis and virulence.

### 4.6. Inhibitors of Morphogenesis and Antifungal Drug Resistance

Interestingly, some small molecules have been identified that are able to both inhibit morphogenesis, as well as block resistance to antifungal drugs, or sensitize drug-resistant fungal lineages. As described above, there are many overlapping cellular pathways in *Candida* that mediate both morphogenesis, as well as resistance to antifungal drugs. As such, identifying drugs that target these pathways may be a powerful strategy for inhibiting virulence and treating drug-resistant fungal infections. For example, recently, it was found that halogenated salicylanilides compounds elicit overexpression of drug resistance genes and are also capable of inhibiting morphogenesis in *C. albicans* [233]. For example, the drug Niclosamide was able to inhibit hyphal formation in both wild-type and drug-resistant *C. albicans* lineages, and transcriptome analysis showed that strains exposed to this drug upregulated genes responsible for drug efflux, such as *CDR1, MDR1* and *TPO3*, while also downregulating genes responsible for filamentous growth. 

The natural product beauvericin has been shown to block the morphogenetic transition between yeast and filamentous growth in *C. albicans* via the transcription factor Brg1, while simultaneously potentiating the activity of the azole antifungals against azole resistant isolates of *C. albicans* and *C. glabrata* [245]. This potentiation of azole activity and sensitization of drug-resistant *Candida* isolates occurs by inhibition of multidrug efflux via the Cdr1 efflux pump [245]. Similarly, the natural product staurosporine enhances the activity of both the azole and echinocandin antifungals [172], and regulates fungal morphogenesis [246]. Interestingly, staurosporine induces, as oppose to represses, *C. albicans* morphogenesis, via cAMP-PKA signaling (Figure 1e) [246]. This is reminiscent of the effect of inhibiting *HSP90* in *C. albicans*, as described above, which both enhances antifungal activity, and induces filamentous growth [72,123,187,254]. Given that Hsp90 is such a highly-connected protein, with many chaperone client proteins and a large interaction network [255], it is likely that depletion of this interconnected protein affects multiple downstream cellular alterations, resulting in pleiotropic effects. For instance, Hsp90 regulates morphogenesis via its interactions with the cAMP-PKA signaling pathway, the cyclin-dependent kinase Cdc28, and other signaling pathways [72,74,256,257]. Through its interaction with the protein phosphatase calcineurin, Hsp90 is able to modulate antifungal drug resistance in *C. albicans* [123,187,188]. Hsp90 also interacts with co-chaperones, such as Sgt1 and Cdc37 [71,258], which impact its role in morphogenesis and drug susceptibility. For example, Cdc37, via its interaction with the kinase Crk1, plays a crucial role in *C. albicans* morphogenesis [259]. Inhibitors which affect both fungal morphogenesis and resistance to antifungal drugs hold great promise as novel therapeutics against fungal infections, or for use in combination with existing antifungal agents to provide more effective treatment of drug-resistant infections.

Finally, the broad-spectrum chelator diethylenetriaminepentaacetic acid (DTPA) was recently discovered to strongly induce filamentation, while also enhancing the susceptibility of *C. albicans* to antifungal drugs [247]. DTPA causes robust filamentation of *C. albicans*, even in non-hyphal inducing cues, by depleting zinc. This filamentation occurs in a manner that is dependent upon the cAMP-PKA signalling pathway, as well as additional transcription factors Brg1 and Rob1 [247]. DTPA also increases fungal susceptibility to echinocandin antifungals and is able to enhance the efficacy of caspofungin against echinocandin-resistant *C. albicans* strains both in vitro and in vivo. DTPA’s role in antifungal susceptibility occurs via chelation of magnesium; it acts to modulate Hog1-MAPK signaling and enhance echinocandin activity [247].

## 5. Conclusions

It is clear that, amongst *Candida* pathogens, there are important connections between antifungal drug treatment, cellular morphogenesis, and resistance to antifungal drugs. It may not be surprising that such a connection exists, given that a majority of antifungal agents target the fungal membrane or cell wall, and that cell wall remodelling is an important component of fungal morphogenesis and the yeast-to-hyphal growth transition. Given the important role of *Candida* filamentation in fungal virulence, and the numerous shared pathways governing filamentation and susceptibility to antifungal agents, targeting these shared signaling pathways may be a powerful strategy to enhance antifungal efficacy while limiting fungal virulence. Indeed, novel anti-virulence compounds that target *Candida* morphogenesis are promising alternatives or complements to the current antifungal arsenal. Future research on fungal signaling pathways that couple morphogenesis with susceptibility to antifungal drugs may help identify new cellular targets for the treatment of life-threatening invasive fungal infections, and the development of novel clinical solutions.

## Figures and Tables

**Table 1:** *Candida* species: morphogenesis, antifungal responses and virulence processes.

Species	Cellular Morphologies	Antifungal Resistance			Virulence Processes
		Azoles	Polyenes	Echinocandins	
*C. albicans*	Yeast, pseudohyphae, and hyphae	S	S	S	Filamentous growth, tissue invasion, adhesion, biofilm formation, hydrolytic enzyme secretion [15,16]
*C. glabrata*	Yeast	I	S	S	Adhesion, biofilm formation, hydrolytic enzyme secretion [17]
*C. tropicalis*	Yeast, pseudohyphae, and hyphae	I	S	S	Filamentous growth, tissue invasion, adhesion, biofilm formation, hydrolytic enzyme secretion [18,19]
*C. parapsilosis*	Yeast, pseudohyphae	S	S	S/I	Tissue invasion, adhesion, biofilm formation hydrolytic enzyme secretion [20,21]
*C. lusitaniae*	Yeast, pseudohyphae	S*	R*	S*	Adhesion, biofilm formation morphogenesis [22,23]
*C. krusei*	Yeast, pseudohyphae	R	S	S	Adhesion, tissue invasion, biofilm formation [24]
*C. auris*	Yeast, pseudohyphae, and hyphae	R*	I*/R*	S*/I*	Filamentation, adhesion, biofilm formation, hydrolytic enzyme secretion [25,26]
*C. guilliermondii*	Yeast, pseudohyphae	R*	S*	R*	Adhesion, biofilm formation, [23,27]
*C. dubliniensis*	Yeast, pseudohyphae and hyphae	S	S	S	Filamentous growth, tissue invasion, adhesion, biofilm formation, hydrolytic enzyme secretion [28,29]

R: resistance, S: susceptible; I: intermediate, based on CLSI and EUCAST breakpoints established for *Candida* species, via [30]. * Indicates species without established breakpoints, where resistance is considered relative to *C. albicans*.

**Table 2:** Compounds that alter *Candida* morphogenesis.

Compound	Mode of Action/Target Pathway	*Candida* Species Tested	Refs.
**Block Filamentation**			
Biatriospora D	Reduction of cAMP signaling via cAMP-PKA; upregulation of farnesol via Dpp3	*C. albicans*	[239]
Sophorolipid	Downregulates hyphal-specific genes	*C. albicans*	[241]
Biaryl compounds	Regulation of Brg1	*C. albicans*	[237]
Filastatin	Downstream of Efg1 (cAMP-PKA) and Cph1 signaling	*C. albicans*	[238]
Niclosamide	Downstream of cAMP-PKA and MAPK signaling	*C. albicans* (filamentation and biofilm formation) and *C. auris* (biofilm formation)	[233]
Tri-Chloro-Salicyanilide (TCSA)	Downstream of cAMP-PKA and MAPK signaling	*C. albicans* (filamentation and biofilm formation) and *C. auris* (biofilm formation)	[233]
6-Gingerol, 6-Shogaol	Downregulates hyphal-specific genes	*C. albicans*	[242]
Bacteriocin EntV	Unknown	*C. albicans*	[243]
Capric acid	Downregulates hyphal-specific genes	*C. albicans*	[244]
Beauvericin	Regulation of Brg1	*C. albicans*	[245]
**Induce Filamentation**			
Staurosporine	Cyr1-PKA signaling	*C. albicans*	[246]
Geldanamycin	Hsp90 and cAMP-PKA signaling	*C. albicans*	[72]
Diethylenetriaminepentaacetic acid (DTPA)	Zinc depletion, cAMP-PKA signaling, Brg1 and Rob1	*C. albicans*	[247]
